# Exploring the acoustic and prosodic features of a lung-function-sensitive repeated-word speech articulation test

**DOI:** 10.3389/fpsyg.2023.1167902

**Published:** 2023-08-30

**Authors:** Biao Zeng, Edgar Mark Williams, Chelsea Owen, Cong Zhang, Shakiela Khanam Davies, Keira Evans, Savannah-Rose Preudhomme

**Affiliations:** ^1^Department of Psychology, University of South Wales, Pontypridd, United Kingdom; ^2^School of Care Sciences, University of South Wales, Pontypridd, United Kingdom; ^3^School of Education, Communication and Language Sciences, Newcastle University, Newcastle upon Tyne, United Kingdom

**Keywords:** speech breathing, COPD, respiration, pause, helicopter task

## Abstract

**Introduction:**

Speech breathing is a term usually used to refer to the manner in which expired air and lung mechanics are utilized for the production of the airflow necessary for phonation. Neurologically, speech breathing overrides the normal rhythms of alveolar ventilation. Speech breathing is generated using the diaphragm, glottis, and tongue. The glottis is the opening between the vocal folds in the larynx; it is the primary valve between the lungs and the mouth, and by varying its degree of opening, the sound can be varied. The use of voice as an indicator of health has been widely reported. Chronic obstructive pulmonary disease (COPD) is the most common long-term respiratory disease. The main symptoms of COPD are increasing breathlessness, a persistent chesty cough with phlegm, frequent chest infections, and persistent wheezing. There is no cure for COPD, and it is one of the leading causes of death worldwide. The principal cause of COPD is tobacco smoking, and estimates indicate that COPD will become the third leading cause of death worldwide by 2030. The long-term aim of this research program is to understand how speech generation, breathing, and lung function are linked in people with chronic respiratory diseases such as COPD.

**Methods:**

This pilot study was designed to test an articulatory speech task that uses a single word (“helicopter”), repeated multiple times, to challenge speech-generated breathing and breathlessness. Specifically, a single-word articulation task was used to challenge respiratory system endurance in people with healthy lungs by asking participants to rapidly repeat the word “helicopter” for three 20-s runs interspersed with two 20-s rest periods of silent relaxed breathing. Acoustic and prosodic features were then extracted from the audio recordings of each adult participant.

**Results and discussion:**

The pause ratio increased from the first run to the third, representing an increasing demand for breath. These data show that the repeated articulation task challenges speech articulation in a quantifiable manner, which may prove useful in defining respiratory ill-health.

## 1. Introduction

Chronic obstructive pulmonary disease (COPD) is the most common long-term respiratory disease. The main symptoms of COPD are increasing breathlessness, a persistent chesty cough with phlegm, frequent chest infections, and persistent wheezing.^1^ There is no cure for COPD, and it is one of the leading causes of death in the world. The principal cause of COPD is tobacco smoking, and estimates indicate that it will become the third leading cause of death worldwide by 2030. This pilot study tested an articulatory speech, which uses a single word “helicopter,” repeated multiple times, to challenge speech-generated breathing and breathlessness.

### 1.1. COPD and lung function

COPD develops slowly, appearing in middle age; initially, it has little effect on lung function, and its impact on lifestyle is minor. As the disease develops, the associated lung dysfunction becomes disabling; the person with COPD becomes increasingly immobile and eventually requires oxygen support. This slow decline can be marked by exacerbations that require acute healthcare intervention. The ability to identify these exacerbations before they occur or early on would improve the quality of life of those with COPD and help reduce healthcare costs.

Severe COPD leads to pronounced breathlessness and alters pulmonary ventilation. These COPD-induced changes subtly affect other breathing-related functions, such as speech articulation and the pause time between words.

A clinical test used to diagnose and stage the severity of COPD is spirometry, which is performed under the supervision of a trained healthcare practitioner. Although speech production is altered by COPD and other lung diseases, signals from speech production have not been used as a diagnostic tool, partially because the changes in voice are subtle.

### 1.2. Speech breathing

The term “speech breathing” is usually used when referring to the manner in which expired air and respiratory mechanics are utilized to produce the airflow necessary for phonation. During speech breathing, a quick inspiration is followed by a prolonged expiration. Quick inspiration can reduce pause time and allows a speaker to retain the floor in a speaking exchange. A volume of air is taken into the lungs and then pushed out through the glottis to enable utterance of speech sounds. The variable amounts of air inhaled are based on the content to be produced.

The cycle of inspiration and expiration in speech breathing is generated using the abdominal muscles, diaphragm, glottis, mouth, and nose. The abdominal muscles (rectus abdominus, external oblique, internal oblique, and transverse abdominus) are located between the ribs and the pelvis on the front of the body. These muscles support the rib cage to expand during inspiration (Hixon et al., 1973). The diaphragm is a large, dome-shaped muscle located at the base of the lungs. When the diaphragm contracts and flattens and the chest cavity enlarges, this contraction creates a vacuum and pulls air into the lungs. Upon exhalation, the diaphragm relaxes and returns to its domelike shape, and air is forced out of the lungs. The abdominal muscles can move the diaphragm and provide more power to empty the lungs. The glottis is the opening between the vocal folds in the larynx; it is the primary valve between the lungs and the mouth, and the sound can be varied by varying its degree of opening.

Usually, speech begins once the lungs have been filled upon the end of inspiration. It therefore begins with a large lung volume (LV), which is associated with longer voice onset times, increased subglottal pressure, increased sound pressure levels, a higher fundamental sound frequency, and increased glottal leakage. In contrast, speech produced at low LVs has been found to be associated with a more adducted vocal state compared with speech produced at high LVs. For instance, Iwarsson et al. (1998) studied the effects of lung volume on the glottal voice source and found that the closed quotient increases with decreasing lung volume, while subglottal pressure, peak-to-peak flow amplitude, and glottal leakage tend to decrease. In addition, Murray et al. (2018) asked speakers to read passages with two speaking voices: typical (baseline and return phases) and breathy vocals (experimental phase). They found that the participants spoke with larger LV excursions during the experimental phase, characterized by increased LV initiation and decreased LV termination compared with the baseline phase.

Regarding the airstream mechanism of speech breathing, many studies have explored the possibility of using speech breathing to predict and diagnose lung function. For specific groups, e.g., patients with asthma or Parkinson's disease, speech measures offer promising monitoring and diagnosis methods. Tayler et al. (2015) reported that healthcare professionals can estimate the predicted forced expiratory volume in one second (FEV_1_ %) based on speech samples from asthma patients. This finding provides evidence that speech is altered in acute asthma.

### 1.3. Effects of age and sex on speech breathing

Across an individual's lifespan, the anatomy of the respiratory system changes, and the functioning of breathing can become limited in association with these changes. For instance, larger bodies typically result in larger lungs and respiratory systems (McDowell et al., 2008). Until age 14, lung length and width both expand linearly (Polgar and Weng, 1979; Zeman and Bennett, 2006). Boys' lungs continue to grow until between the ages of 18 and 20, while girls' lung growth patterns settle at ~14 years of age (Polgar and Weng, 1979). However, men and women typically start to lose weight beyond the age of 60, and this loss continues into the seventh and eighth decades of life. Lung volume, static recoil pressure, and respiratory muscle strength all undergo physiological changes because of the respiratory system. Along with anatomical and physiological changes in the respiratory system, speech breathing patterns and features change across the lifespan.

Vocal intensity is an acoustic measure. During everyday speech production, speakers have to raise their vocal intensity to ensure that they are heard in noisy environments. To increase vocal intensity, the respiratory system will generate higher subglottal air pressures (Finnegan et al., 2000). There are differences between age groups in terms of the way in which intensity is increased during speech production: children, teenagers, and young adult speakers can use larger lung and rib cage volume excursion to increase intensity, but older adults do not show the same pattern. Utterance length is a measure of the linguistic (prosodic) feature of speech, and there is a significant correlation between utterance length and respiratory function. Utterance length is defined as the number of syllables or words produced in one speech breath.

Speech breathing patterns change with age, but little consideration has been given to sex-based differences within COPD clinical research (Somayaji and Chalmers, 2022). There is, however, emerging and considerable evidence to suggest that sex contributes to disease pathogenesis, risk, diagnosis, prevalence, severity, and clinical outcomes (Fletcher and Peto, 1977; Doyal, 2001; Carey et al., 2007; Townsend et al., 2012). In addition, there have been calls for researchers to better understand the mechanisms underpinning these observed differences (Silveyra et al., 2021). Despite these calls, the literature is scant, but it points to anatomical differences between sexes and the influence of sex hormones, the menstrual cycle, and other diseases (e.g., asthma), which are said to modulate these sex differences in COPD (LoMauro and Aliverti, 2018). For example, standard morphometric measures have shown that males have larger lungs than females (Thurlbeck, 1982). In addition, females have smaller airway diameters and lung volume, resulting in lower peak expiratory flow than males. Furthermore, respiratory symptoms in females (e.g., wheezing, dyspnea, and cough) vary significantly with menstrual cycle-induced hormonal changes: specifically, these COPD symptoms tend to get worse in the mid-phase of the cycle (Macsali et al., 2012). Understanding the contribution of sex and gender to COPD will help with the development of precision medicine and the effective daily management of COPD.

### 1.4. Three-tier feature measures

Speech breathing is a special breathing function. It is a multi-faceted phenomenon integrating breathing, speech production, and articulation. Distinct types of information can be extracted from the utterance of speech sounds by examining the characteristics of speech, e.g., acoustic features, prosodic features, and certainly breathing-related features. Therefore, we developed a systematic analysis method with three tiers of feature measures. The three tiers consist of acoustics, prosody, and breathing. Acoustics refers to the physical properties of sounds, and this measure captures speech-related information, e.g., vowel formants, intensity (perceived as loudness), or fundamental frequency (perceived as pitch). Prosodic features, in this study, refers to how speech sounds are organized, including length of run and pause ratio. Measures of breathing features, specifically in relation to speech breathing, are under development. People usually take around 10–15 breaths per minute when resting. This is described as the respiratory rate. In the current study, we adopted respiratory rate as the key measure of breathing.

Vocal intensity is the most widely investigated acoustic feature in studies of speech breathing. Speakers use larger lung and rib cage volume excursions when increasing their vocal intensity (Stathopoulos and Sapienza, 1997). Further studies on prosodic information have revealed the correlation of these measures with respiratory functions. For instance, studies have revealed a correlation between utterance length and respiratory function (e.g., Sperry and Klich, 1992; Whalen and Kinsella-Shaw, 1997). Age-related effects also occur, with older adults producing shorter utterances than young adults do. Huber (2008) examined age-related changes in speech breathing by measuring utterance length and loudness, and found that age-related effects increased as utterances became longer. These results suggest that older adults have a more challenging time when the speech system is being taxed by both utterance length and loudness. The data were also consistent with the hypothesis that both young and older adults use utterance length in premotor speech planning processes.

A wide range of speech features have been judged relevant for and investigated in relation to health status. Farrús et al. (2021) proposed two types of speech features, acoustic and prosodic information, and applied them for the detection and classification of bipolar disorder. They argued that prosodic information, which is conveyed through intonation, stress, and rhyme, could reflect the emotional aspects of the individual. In this study, we investigated acoustic and prosodic features and focused on the prosodic information.

In recent years, computerized deep learning methods have offered new ways of modeling speech and analyzing it for healthcare applications (Cummins et al., 2018). For instance, Nallanthighal et al. (2021) proposed using deep learning to investigate breathing. Breathing (i.e., inhalation and expiration) is essential and these are the primary mechanisms driving speech production. These authors explored techniques for sensing the breathing cycle and extracting breathing metrics from speech using deep learning architectures, and addressed the challenges involved in establishing the usefulness of applying this technology. Estimating breathing patterns from speech provides information about the corresponding respiratory parameters, which would enable assessment of the speaker's respiratory health using speech alone.

Specifically, in the present study, pause ratio was measured as one key feature of rhythm. Fuchs and Rochet-Capellan (2021) reviewed the respiratory foundations of spoken language and highlighted the fact that breathing interacts with respiration, syntax, and planning. We should distinguish respiratory and linguistic pauses in a breathing cycle. A typical respiratory pause occurs during a breathing cycle. In a normal breathing cycle at rest, there is an in-breath (inhalation) followed by an out-breath (exhalation). The out-breath is followed by an automatic pause (or period of no breathing) lasting ~1 to 2 s. In contrast, a linguistic pause is a silent pause or filled pause containing *um* or *uh*. In terms of speech breathing, an in-breath may play the role of such a linguistic pause, which can inspire speech and empower the following articulation. In the following section, we introduce the potential use of prosodic information as a measure of lung function and analyse the potential correlation between prosodic features and lung function.

In the present study, a speech breathing task, namely the “helicopter task,” was designed to measure the acoustic, prosodic, and breathing characteristics of speech. The helicopter task requires participants to repeat the word “helicopter” as quickly as possible for 20 s, followed by a 20-s break of silence. This was repeated twice, creating a task consisting of three runs lasting ~100 s in total. Based on previous studies, acoustic features include a wide range of parameters, e.g., frequency and vocal intensity. Vocal intensity is of specific interest. Utterance length is the key umbrella concept of prosodic features; in this study, it was calculated in terms of speech rate and word duration. A pause, as a critical parameter for measurement of speech breathing, was defined as a silence filled with no utterance of the word “helicopter” and measured in terms of pause ratio. The pause ratio was calculated as the duration of the pause divided by the entire 20-s duration of word repetition.

Three main research hypotheses were addressed:

1. Run effect: It was assumed that, with airflow consumed over the course of the task, prosodic measures would be affected: in particular, pauses would become longer, and, correspondingly, pause ratio would increase and more breaths would be taken in the later runs.
2. Sex differences: It was predicted that female speakers would produce higher-frequency speech, lower-intensity speech, shorter syllable durations, and longer pause ratios compared to male speakers.
3. As the three tiers of features included a wide range of variables in the study, we predicted that acoustic and prosodic features would be significant predictors of measures of lung function. A multiple regression method was employed to explore these predictors.

## 2. Materials and methods

### 2.1. Participants

A total of 27 healthy, native English-speaking participants (12 men, 15 women; mean age: 26, range 19–55 years; height: 1.68 ± 0.12 m; weight: 68.8 ± 14.0 kg, *n* = 24) were recruited from the University of South Wales community through random sampling. Two participants did not follow the instructions, and their data were not included in the analysis. All participants filled out the Clinical Report Form One (Appendix 1), which consisted of seven questions: age, sex, height, weight, respiratory condition, smoking history, and breathing status. No other general health status, medication, or physical activity parameters were investigated.

Predicted lung function was calculated using the Global Lung Function Initiative index (European Respiratory Society). Two measures of forced expired volume (FEV1) and forced vital capacity (FVC) were predicted from weight and height data. A *t*-test showed that the means of FEV1, FVC, and FEV1/FVC ratio were statistically different between sexes (*p*-values < 0.01).

The study was approved by the Faculty of Life Science and Education Ethics Panel, University of South Wales (No 210901HR), in accordance with the Declaration of Helsinki. Written and verbal informed consent were obtained from each participant.

### 2.2. Protocol

The study used a speech articulation task designed to test lung health. The design was derived from that of the diadochokinesis (DDK) task, which is one of the oldest and most frequently used tasks for evaluating various types of speech communication problems. It often involves fast repetition of single words or of non-speech oral movements such as opening and closing of the lips. It has numerous variations and is also referred to as verbal, oral, or phonoarticulatory DDK. It has cross-disciplinary applications in areas such as aging, biomedical engineering, biological sciences, communication sciences and disorders, computational methods in biomedicine, craniofacial surgery, dentistry, neurology and neurosciences, and oral surgery (Kent et al., 2022).

In the current study, we used a task involving the repetition of a single polysyllabic word to explore its potential in measuring lung function. This word “helicopter” was chosen from a list of words commonly used in speech therapy testing. This list is shown in Table 1. In this list, the words “ambulance,” “vegetable,” and “animals” can be pronounced either with a mid-central vowel, the schwa /ǝ/, or without. The uncertainty of the presence of the schwa could result in changes in syllable structure, duration, loudness, and other aspects of articulation. In order to keep all measurements consistent and accurate, these words were not chosen for the current task. Among the rest of the words, “hippopotamus” and “helicopter” contain the voiceless glottal fricative /h/, which requires the maximum amount of airflow to maintain articulation compared with the consonants in the other words, which are mostly stops, nasals, or approximants. Between the two words containing /h/, “hippopotamus” is a low-frequency word and may prevent speakers from articulating fluently. Therefore, “helicopter” was chosen as the word for our task.

**Table 1:** Phonemes in ten common words used in speech therapy tests.

**Word**	**Number of syllables**	**IPA (International Phonetic Alphabet)**
Ambulance	3	/'ambjƱl(ǝ)ns/
Hippopotamus	5	/hɪpǝ'pɐtǝmǝs/
Computer	3	/kǝm'pju:tǝ/
Spaghetti	3	/spǝ'gεti/
Vegetables	3	/'vεdʒtǝb(ǝ)l/z//
Helicopter	4	/hεlɪkɐptǝ/
Animal	3	/'anɪm(ǝ)l/
Caravan	3	/'karǝvan/
Caterpillar	4	/katǝpɪlǝ/
Butterfly	3	/'bʌtǝfl/ai//

### 2.3. Procedures

Each participant was asked to sit still in a quiet room at a distance of ~40 cm from their computer. Their task was to produce the word “helicopter” as quickly as possible and as many times as possible within a 20-s production period. Each participant was given three 20-s production periods with a 20-s forced break between the first and second production periods and between the second and third production periods. The experimenter gave a signal for the start of each production period and the start of each break. The instructions were as follows:

“*For this exercise, you will be asked to repeat the word ‘helicopter,' as before, but this time for only 20 s at a time. For this exercise, I want you to do this three times but with a short break in between. When I say ‘go,' please start repeating ‘helicopter' until I say ‘stop.' After a short interval I will say ‘go,' so like before keep going until I say ‘stop.' After another short rest, I will ask you to do this one more time. I will time and record the whole exercise, so please sit quietly during the two resting intervals.”*

“*Do you have any questions you would like to ask?”*

“*Are you ready to begin?”*

The recordings were made online via Microsoft Teams, with both the experimenter and the participant in the session. During testing, the camera was switched off and only speech was recorded. Only participants' speech was analyzed.

The audio recordings were converted into.wav files using the audio editing software package Audacity. The three 20-s runs were extracted as separate files for acoustic analysis. Only instances of the word produced in full were kept for analysis; instances with disfluencies, prominent background noise, or overlap with the experimenter's instructions were excluded from acoustic analysis. Audible breaths (inspirations) in each run were identified and matched to the digital recording, allowing the number of breaths to be counted and the duration of each breath to be calculated. Word boundaries and pause boundaries were manually segmented in all audio files in Praat (Boersma and Weenink, 2022) by a first annotator; a second annotator, who was a trained phonetician, then checked the boundaries and made corrections to the annotation.

For the acoustic analysis, F0 and formants were both extracted as mean values across each word as a whole, using a Praat script. F0 data were extracted with a range of 60–500 Hz, and the formants were extracted with a ceiling of 5,000 Hz. Mean F1 and mean F2 values were measured with a ceiling of 5,000 Hz. Intensity data were extracted using a minimum F0 of 60 Hz, with a 0.01 step. The length of pauses was measured, including only periods when the speaker was taking a breath between words; the initial and final silence periods at the start and end of each production period were excluded. The pause ratio was the total duration of the pauses in the production period divided by the total duration of the production period from the production of the first instance.

### 2.4. Statistical analysis

Means ± SD are reported. Acoustic features (e.g., intensity, F0, F1, F2, and F0 range) and prosodic features, e.g., speech rate (number of words per second for the entire run), word duration (mean duration for the word “helicopter” across the entire run), and pause ratio (mean pause duration for the entire run), are reported in Table 2. As the lung growth pattern stabilizes at 13–14 years of age for females and 18–20 years for males (Polgar and Weng, 1979), age is not a factor that we aimed to investigate in the study. Instead, the two independent variables in this study were sex and run (the repetition order in the task). A two-way repeated measure ANOVA (analysis of variance) was conducted to test for the effects of these variables on acoustic, prosodic, and breathing measures. If there was an effect of run on any feature, a regression was conducted to predict the effect on the lung function measures.

**Table 2:** Comparison of voice characteristics between male and female speakers.

**Mean**		**Female**		**Male**	
		**Mean**	**SD**	**Mean**	**SD**
Height (cm)		161.00	9.18	176.60	8.90
Weight (kg)		58.67	8.72	79.80	11.41
Predicted FEV1 (liters)		3.31	0.46	4.53	0.36
Predicted FVC (liters)		3.83	0.58	5.45	0.40
FEV1/FVC ratio		0.88	0.02	0.84	0.03
Intensity (dB)	Run 1	66.52	6.65	68.70	4.50
	Run 2	67.87	6.48	69.23	3.71
	Run 3	67.64	6.20	68.47	3.73
F0 (Hz)	Run 1	194.39	28.22	133.27	10.85
	Run 2	195.94	37.01	137.84	15.39
	Run 3	198.39	30.76	141.76	8.36
F1 (Hz)	Run 1	762.62	81.90	722.10	73.01
	Run 2	752.83	89.48	726.87	81.66
	Run 3	769.09	88.08	754.37	89.28
F2 (Hz)	Run 1	1868.28	148.26	1826.54	174.60
	Run 2	1868.10	123.51	1843.20	151.93
	Run 3	1887.05	141.15	1865.62	133.49
F0 range (Hz)	Run 1	89.08	47.66	154.49	106.14
	Run 2	80.42	41.09	187.64	98.44
	Run 3	101.17	44.23	191.69	82.15
Speech rate (number of words per second)	Run 1	1.47	0.20	1.72	0.22
	Run 2	1.53	0.19	1.69	0.26
	Run 3	1.47	0.17	1.67	0.26
Word duration (milliseconds)	Run 1	164	21	140	19
	Run 2	157	18	139	22
	Run 3	159	18	142	20
Pause ratio (%)	Run 1	6.5	3.1	5.4	3.7
	Run 2	5.9	2.8	8.9	3.7
	Run 3	8.0	2.3	9.1	4.0

## 3. Results

### 3.1. Voice characteristics: acoustic and prosodic features

Among the acoustic features, mains effect of run were found on intensity and F1. Specifically, a two-way repeated measures ANOVA showed a significant main effect of run on intensity, *F* (2.38) = 5.35, *p* = 0.009, $\eta_{\text{p}}^{2}$ = 0.220. Pairwise comparison showed that the intensity of the first run (Mean = 67.61, SE = 1.34) was significantly lower than that of the second run (Mean = 68.55, SE = 1.26), *p* < 0.001. It is intriguing that a two-way repeated measures ANOVA also showed a significant main effect of run on F1, *F* (2.38) = 5.53, *p* = 0.008, $\eta_{\text{p}}^{2}$ = 0.225. Pairwise comparison showed that F1 was significantly higher for the third run (Mean = 761.73, SE = 19.90) than for the first run (Mean = 742.36, SE = 17.69, *p* = 0.018) and the second run (Mean = 739.85, SE = 19.48, *p* = 0.006), but there was no difference between the first and second runs in terms of F1.

Main effects of sex on F0 and F0 range were observed. Specifically, a two-way repeated measures ANOVA showed a significant main effect of sex on F0, *F* (1.19) = 25.21, *p* < 0.001, $\eta_{\text{p}}^{2}$ = 0.570. Pairwise comparison showed that F0 was significantly higher in female speakers (Mean = 196.24, SE = 7.21) than in male speakers (Mean = 137.62, SE = 9.19), which is consistent with previous studies. A two-way repeated measures ANOVA also showed a significant main effect of sex on F0 range, *F* (1.18) = 9.49, *p* = 0.006, $\eta_{\text{p}}^{2}$ = 0.345. Pairwise comparison showed that F0 range was significantly wider in male speakers (Mean = 177.94, SE = 22.96) than in female speakers (Mean = 90.22, SE = 16.85).

With regard to prosodic features, there was a significant main effect of run on pause ratio, *F* (2.36) = 5.26, *p* = 0.010, $\eta_{\text{p}}^{2}$ = 0.226. Pairwise comparison showed that the third run was associated with the highest pause ratio (Mean = 0.085, SE = 0.007), and that this was significantly higher than that of the first run (Mean = 0.059, SE = 0.008, *p* = 0.014), but not the second run. There was no significant difference between the second and third runs.

A main effect of sex on speech rate was also observed, *F* (1.19) = 4.85, *p* < 0.001, $\eta_{\text{p}}^{2}$ = 0.203. The interaction between sex and run was significant, *F* (2.38) = 3.83, *p* = 0.031, $\eta_{\text{p}}^{2}$ = 0.168. Pairwise comparison showed that, in the first run only, male speakers articulated the word “helicopter” more quickly than female speakers (men: Mean = 1.72, SE = 0.07 vs. women: Mean = 1.47, SE = 0.06, *p* = 0.012).

A multiple regression analysis was conducted to explore which features contribute significantly to lung function, specifically FEV1 and FVC. Based on the ANOVA results, we selected intensity, F1, and pause ratio for inclusion as predictor variables. The two criterion variables were FEV1 and FVC. A multiple regression with backward elimination was conducted in SPSS for each run. Using backward elimination, we attempted to only include significant predictor variables in the regression model.

The regression results showed that the regression model was significant for the criterion variable of FEV1 only in the case of the second run, *F* (1.16) = 6.23, p = 0.025; pause ratio was the only significant predictor included, adjusted *R*-squared = 0.246, Beta = 0.542, *p* = 0.025. For the first and third runs, none of the predictors tested (intensity, F1, and pause ratio) was a significant predictor. A similar pattern occurred for FVC. A significant regression model only emerged in the case of the second run, *F* (1.16) = 6.61, *p* = 0.021, where pause ratio was the only significant predictor, adjusted *R*-squared = 0.26, Beta = 0.553, *p* = 0.021.

### 3.2. Breathing characteristics

All three runs for each participant (*n* = 26) were analyzed (a total of 168 breathes, split between men and women at a ratio of 85:83). For four participants, no inspirations were recorded.

The mean duration of the inspirations taken while completing the three “helicopter” runs was 0.283 ± 0.161 s (range: 0.04–0.88 s), *n* = 168, with no differences observed between the sexes. There were also no statistically significant differences (*p* = 0.149) between runs in terms of duration or number of inspirations. The occurrence of the first inspiration was significantly earlier in the second run compared to the first (Run 1: 59 ± 19%; Run 2: 43 ± 15 %; Run 3: 49 ± 22 %).

**Figure 1:**
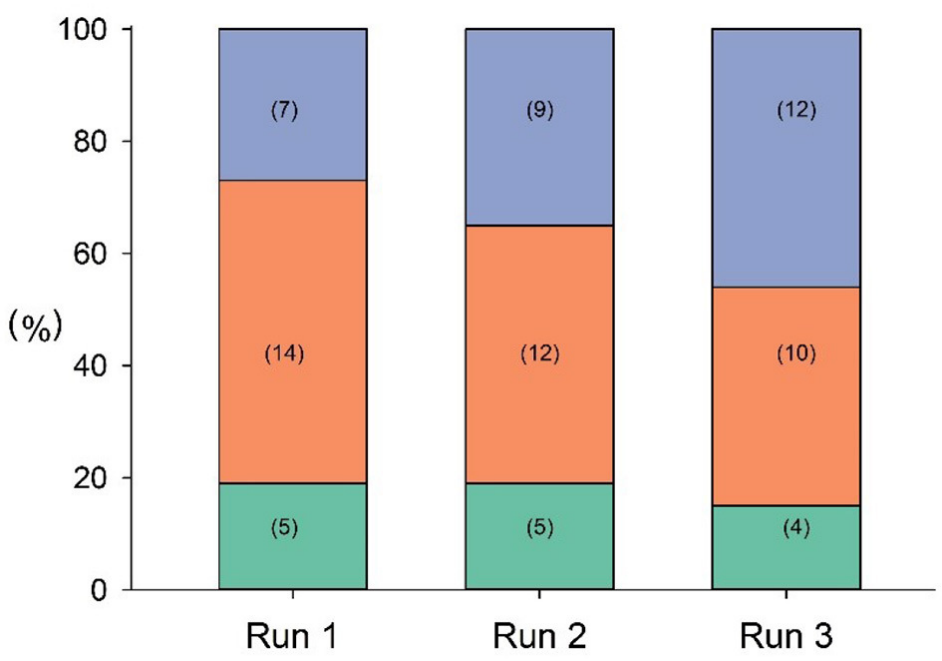
Frequency of inspiration in each run (green: no inspirations detected; orange: 1, 2 inspirations; purple: 3+ inspirations). Number of cases is shown in brackets.

In the first run, approximately half (14/26) of the participants took 1–2 inspirations, but by the third run, this had risen to more than 3 inspirations (12/26) (Figure 1).

## 4. Discussion

In summary, the effects of both sex and run number on different acoustic features, prosodic features, and breathing measures were investigated in this study. Effects of sex occurred for F0, F0 range, and speech rate. Female speakers showed higher F0 values than male speakers, which is consistent with previous studies. Male speakers articulated the word “helicopter” more quickly than female speakers, but only during the first run, which might suggest that their speech rate decreased as articulation load increased. However, the current study also found that male speakers showed a broader F0 range than female speakers. This result differs from those of some previous studies, and further investigation should be considered.

Effects of run number on intensity, F1, and pause ratio were also observed in the present study. As more runs were taken, intensity and F1 increased among male and female speakers. Correspondingly, as intensity and F1 increased with each run, the speakers took more inspirations, which was indicated by the increase in pause ratio over the course of the three runs. The speakers needed to take more pauses to inhale air as they articulated the words in the later (second and third) runs. The mechanism underlying the effect of run on intensity needs further investigation. Huber et al. (2005) proposed three means of increasing speech loudness: increased recoil pressures, increased expiratory tension, and a combination of both. Unlike their design, in which the speaker was requested to increase loudness, the current “helicopter” task is deliberately designed to induce respiratory load by requiring participants to repeat words quickly over the course of three runs. We need to clarify which approach was adopted by speakers to increase intensity and F1. In addition, we need to clarify whether the increase in air intake was used to increase intensity or to compensate for respiratory load or physiological fatigue.

The multiple regression results indicated that pause ratio was the sole significant variable to predict two lung function measures, FEV1 and FVC, and did so only on the second run. The analysis of breathing characteristics showed that the first inspiration occurred earliest in the second run, among all three runs. The correlation of pause ratio and inspiration indicates that the relationship between run number and inspiration in speech breathing might not be linear. Essentially, to understand this finding, the mechanism underlying the use of pauses in speech breathing should be interpreted in relation to whether it represents an inhaling process or is mixed with another breathing event, for instance, breathiness.

In the present study, three tiers of features have been proposed to extract and categorize rich information from speech breathing and to provide insight into the relevance of factors on each of these tiers to lung function. In the current data analysis, for instance, the weights and roles of acoustic, prosodic, and breathing features are one issue that calls for more work. Even within the category of acoustic features, the changes in various features and measures that could be attributed to speech breathing patterns or loads are not clear. Previous studies have investigated the roles of F0, F1, and F2. Lively et al. (1993) found that, in a workload condition, talkers produced utterances with increased amplitude and amplitude variability, decreased spectral tilt and F0 variability, and increased speaking rate. However, no changes in F1, F2, or F3 were observed across conditions. In contrast, Huttunen et al. (2011) studied the utterances of 13 male military pilots that were recorded during simulated combat flights, and found that the strongest associations were observed between three types of cognitive load and F1 and F2 changes in back vowels.

The present study provides empirical evidence for the use of acoustic and prosodic features of speech as health sensors and indicators. Specifically, the repeated articulation “helicopter” task and the pause ratio measure are sensitive to changes in speech breathing and reflect lung function. Within the range of other available acoustic and prosodic features, we need to further screen for sensitive and specific indicators and investigate their mechanistic link with lung function. Our single-word-based articulation task may potentially represent a rapid tool for prediction of lung health in people with COPD. Therefore, the use of speech breathing and relevant linguistic–prosodic information could be further integrated into future home-based healthcare systems.

## Data availability statement

The raw data supporting the conclusions of this article will be made available by the authors, without undue reservation.

## Author contributions

BZ and EW: original idea, original draft, data analysis, and writing. CZ: original speech data analysis and writing. SD and CO: writing and review. BZ, CO, KE, and S-RP: original data collection. All authors approved the final version of the article.

## Footnotes

^1^ https://www.nhs.uk/conditions/chronic-obstructive-pulmonary-disease-copd/

## In memoriam

It is sad that Prof. Edgar Mark Williams passed away prior to the publishing of the research paper. This paper is in a deep and sincerely memory for Prof. Williams' research innovation, energy, curiosity and ambition in breathing.

## References

[B1] Boersma P. Weenink D. (2022). Praat: Doing Phonetics by Computer [Computer Program]. Version 6, 3.01. Available online at: http://www.praat.org/

[B2] Carey M. A. Card J. W. Voltz J. W. Arbes S. J. Jr. Germolec D. R. Korach K. S.. (2007). (2007). It's all about sex: gender, lung development and lung disease. Trends Endocrinol. Metabol. 18, 308–313. 10.1016/j.tem.08003 17764971 PMC2391086

[B3] Cummins N. Baird A. Schuller B. W. (2018). Speech analysis for health: current state-of-the-art and the increasing impact of deep learning. Methods 151, 41–54. 10.1016/j.ymeth.07007 30099083

[B4] Doyal L. (2001). Sex, gender, and health: the need for a new approach. BMJ 323, 1061–1063. 10.1136/bmj.323.7320.1061 11691769 PMC1121552

[B5] Farrús M. Codina-Filbà J. Escudero J. (2021). Acoustic and prosodic information for home monitoring of bipolar disorder. Health Informatics J. 27, 1460458220972755. 10.1177/1460458220972755 33438502

[B6] Finnegan E. M. Luschei E. S. Hoffman H. T. (2000). Modulations in respiratory and laryngeal activity associated with changes in vocal intensity during speech. J. Speech Lang. Hear. Res. 43, 934–950. 10.1044/jslhr.4304.934 11386480

[B7] Fletcher C. Peto R. (1977). The natural history of chronic airflow obstruction. Br. Med. J. 1, 1645–1648. 10.1136/bmj.1.6077.1645 871704 PMC1607732

[B8] Fuchs S. Rochet-Capellan A. (2021). The respiratory foundations of spoken language. Ann. Rev. Ling. 7, 13–30. 10.1146/annurev-linguistics-031720-10390732299308

[B9] Hixon T. J. Goldman M. D. Mead J. (1973). Kinematics of the chest wall during speech production: volume displacements of the rib cage, abdomen, and lung. J. Speech Hear. Res. 16, 78–115. 10.1044/jshr.1601.78 4267384

[B10] Huber J. E. (2008). (2008). Effects of utterance length and vocal loudness on speech breathing in older adults. Respiratory Physiol. Neurobiol. 164, 323–330. 10.1016/j.resp.08007 18790093 PMC2636560

[B11] Huber J. E. Chandrasekaran B. Wolstencroft J. J. (2005). Changes to respiratory mechanisms during speech as a result of different cues to increase loudness. J. Appl. Physiol. 98, 2177–2184. 10.1152/japplphysiol.01239.2004 15705723 PMC2657603

[B12] Huttunen K. H. Keränen H. I. Pääkkönen R. J. Päivikki Eskelinen-Rönkä R. Leino T. K. (2011). Effect of cognitive load on articulation rate and formant frequencies during simulator flights. J. Acoust. Soc. Am. 129, 1580–1593. 10.1121/1.3543948 21428521

[B13] Iwarsson J. M. Thomasson M. Sundberg J. (1998). Effects of lung volume on the glottal voice source. J. Voice 12, 424–433. 10.1016/S0892-1997(98)80051-9 9988029

[B14] Kent R. D. Kim Y. Chen L. M. (2022). Oral and laryngeal diadochokinesis across the life span: a scoping review of methods, reference data, and clinical applications. J. Speech Lan. Hearing Res. 65, 574–623. 10.1044/2021_JSLHR-21-00396 34958599

[B15] Lively S. E. Pisoni D. B. Van Summers W. Bernacki R. H. (1993). Effects of cognitive workload on speech production: acoustic analyses and perceptual consequences. J. Acoust. Soc. Am. 93, 2962–2973. 10.1121/1.405815 8315159 PMC3499954

[B16] LoMauro A. Aliverti A. (2018). Sex differences in respiratory function. Breathe 14, 131–140. 10.1183/20734735.000318 29875832 PMC5980468

[B17] Macsali F. Svanes C. Bjørge L. Omenaas E. R. Real F. G. (2012). Respiratory health in women: from menarche to menopause. Expert Rev. Resp. Med. 6, 187–202. 10.1586/ers.12.15 22455491

[B18] McDowell M. A. Fryar C. D. Ogden C. L. Flegal K.M. (2008). Anthropometric reference data for children and adults: United States, 2003–2006. Natl. Health Stat. Report 22, 1–48. 10.1037/e623932009-001 25585443

[B19] Murray E. S. H. Michener C. M. Enflo L. Cler G. J. Stepp C. E. (2018). The impact of glottal configuration on speech breathing. J. Voice 32, 420–427. 10.1016/j.jvoice.07001 28838793 PMC6062009

[B20] Nallanthighal V. S. Mostaani Z. Härmä A. Strik H. Magimai-Doss M. (2021). Deep learning architectures for estimating breathing signal and respiratory parameters from speech recordings. Neural Networks 141, 211–224. 10.1016/j.neunet.03029 33915446

[B21] Polgar G. Weng T. R. (1979). The functional development of the respiratory system: from the period of gestation to adulthood. Am. Rev. Resp. Dis. 120, 625–695. 384853 10.1164/arrd.1979.120.3.625

[B22] Silveyra P. Fuentes N. Rodriguez Bauza D. E. (2021). Sex and gender differences in lung disease. Lung Inflammation in Health and Disease, Volume II. (Cham: Springer), (pp. 227-258). 10.1007/978-3-030-68748-9_14 PMC8221458 34019273

[B23] Somayaji R. Chalmers J. D. (2022). Just breathe: a review of sex and gender in chronic lung disease. Eur. Resp. Rev. 31, 21. 10.1183./16000617.0111-2021 35022256 PMC9488531

[B24] Sperry E. E. Klich R. J. (1992). Speech breathing in senescent and younger women during oral reading. J. Speech Lang. Hear. Res. 35, 1246–1255. 10.1044/jshr.3506.1246 1494270

[B25] Stathopoulos E. T. Sapienza C. M. (1997). Developmental changes in laryngeal and respiratory function with variations in sound pressure level. J. Speech Lang. Hear. Res. 40, 595–614. 9210117 10.1044/jslhr.4003.595

[B26] Tayler N. Grainge C. Gove K. Howarth P. Holloway J. (2015). Clinical assessment of speech correlates well with lung function durnig induced bronchoconstriction. NPJ Primary Care Resp. Med. 25, 1–3. 10.1038/npjpcrm.2015.6 25719976 PMC4373502

[B27] Thurlbeck W. M. (1982). Postnatal human lung growth. Thorax 37, 564–571. 10.1136/thx.37.8.564 7179184 PMC459376

[B28] Townsend E. A. Miller V. M. Prakash Y. S. (2012). Sex differences and sex steroids in lung health and disease. Endo. Rev. 33, 1–47. 10.1210/er.2010-0031 22240244 PMC3365843

[B29] Whalen D. H. Kinsella-Shaw J. M. (1997). Exploring the relationship of inspiration duration to utterance duration. Phonetica 54, 138–152. 10.1159/000262218 9396165

[B30] Zeman K. L. Bennett W. D. (2006). Growth of the small airways and alveoli from childhood to the adult lung measured by aerosol-derived airway morphometry. J. Appl. Physiol. 100, 965–971. 10.1152/japplphysiol.00409.2005 16357074

