# Supplementary material for: Exploring the acoustic and prosodic features of a lung-function-sensitive repeated-word speech articulation test
Source: Front Psychol. 2023 Aug 30;14:1167902. doi: 10.3389/fpsyg.2023.1167902 (PMC10499508; doi:10.3389/fpsyg.2023.1167902)
Supplement: Supplementary file 1 [file Data_Sheet_1.docx]

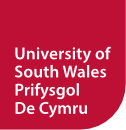
 Clinical Report Form One (CRF1)

Study Title: Exploring the relationship between Speech Articulation and Lung function in Obstructive Lung Disease: a feasibility study.

To be completed by researcher.

Study Pin:

1) Age (years): _____

2) Sex. M/F or Gender. Specified: ___________. Not Specified___________

3) Reported Height: _____ feet/metres

4) Reported weight: _____ stones/kilograms

5) Any known long term respiratory condition (Eg: Asthma): Yes/No

If yes, what is the condition? ______________________

6) Do you smoke or an ex-smoker? Yes/No

If yes. Are you a current smoker: How often do you smoke per day: _______________________

Or

When did you give up smoking? Approximately how much did you smoke per day? __________

7) Do you suffer bouts of breathlessness: infrequently (once a day), frequently (2-3 per day) or often (several times per day)?

Details: _____________________________________________________

Date Completed:

Initial of completing researcher:
